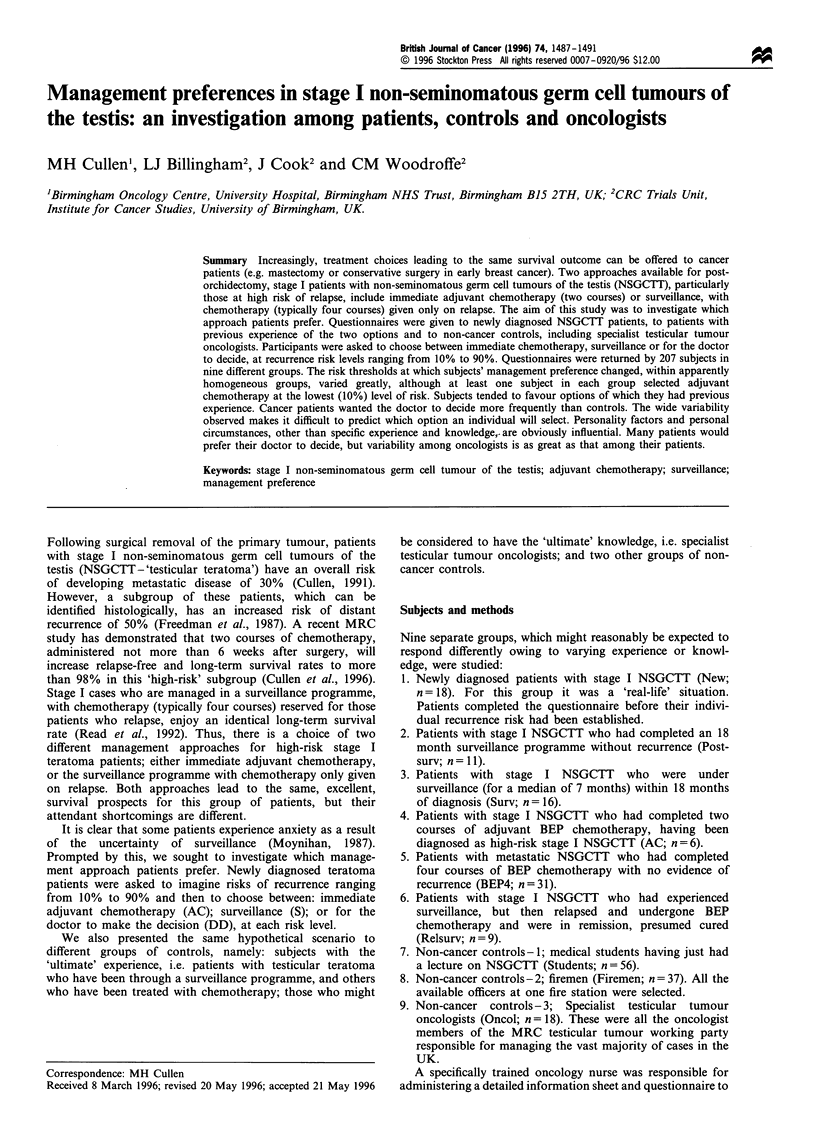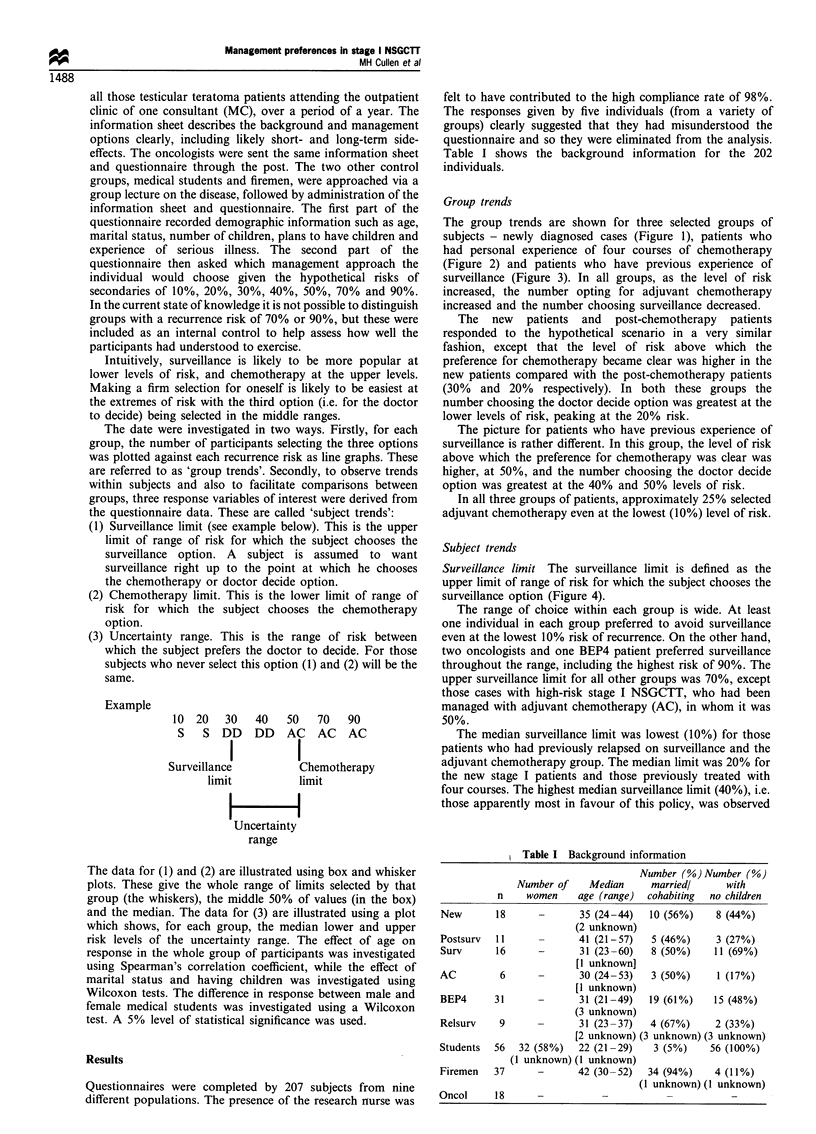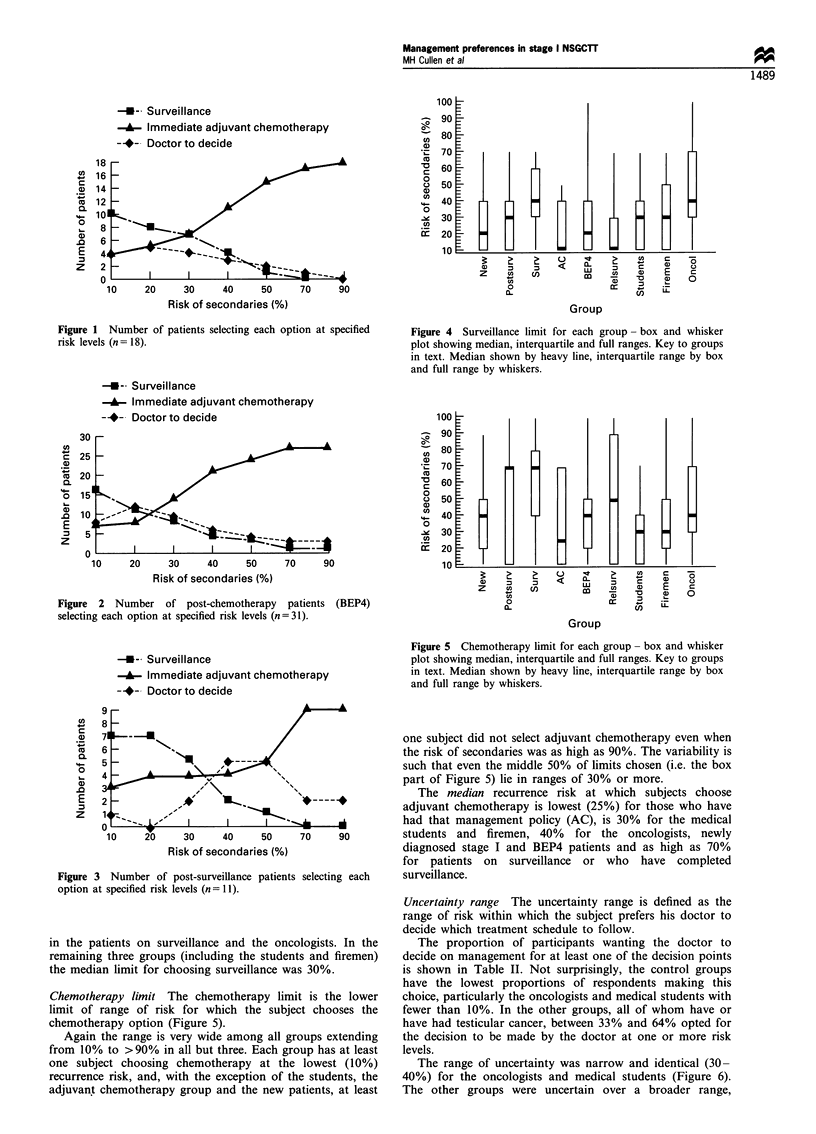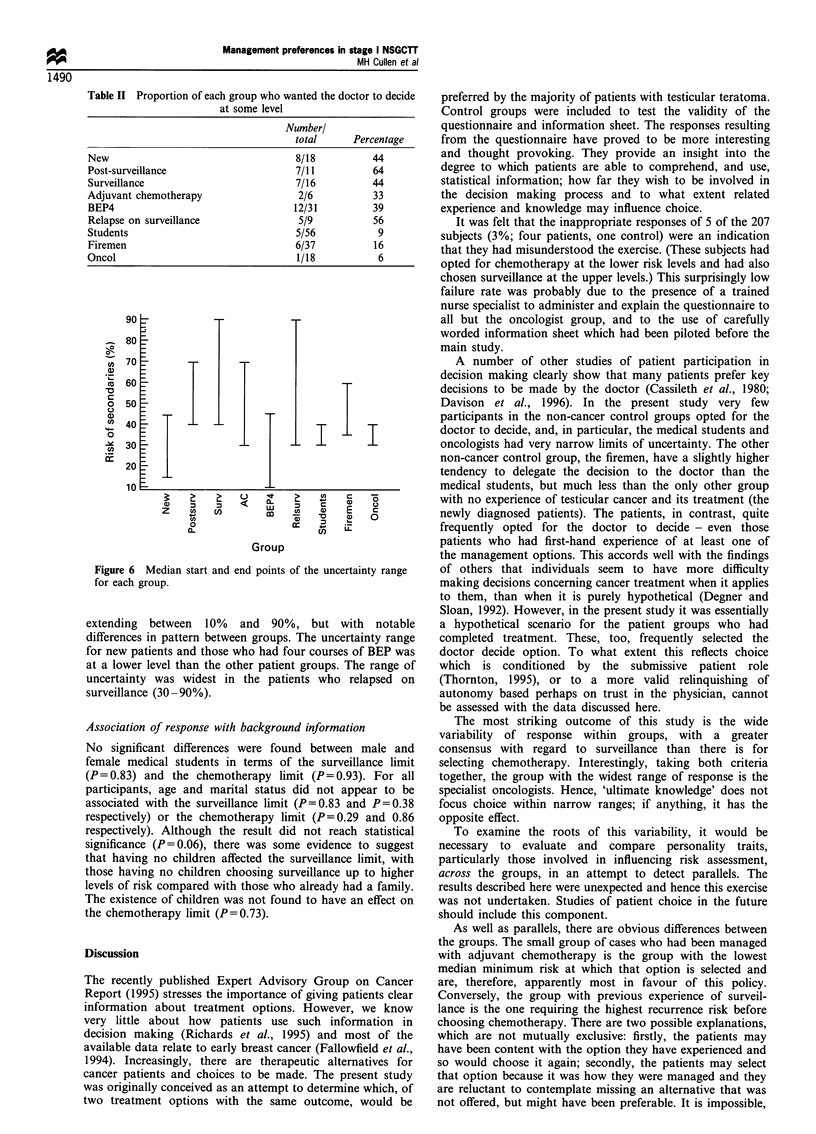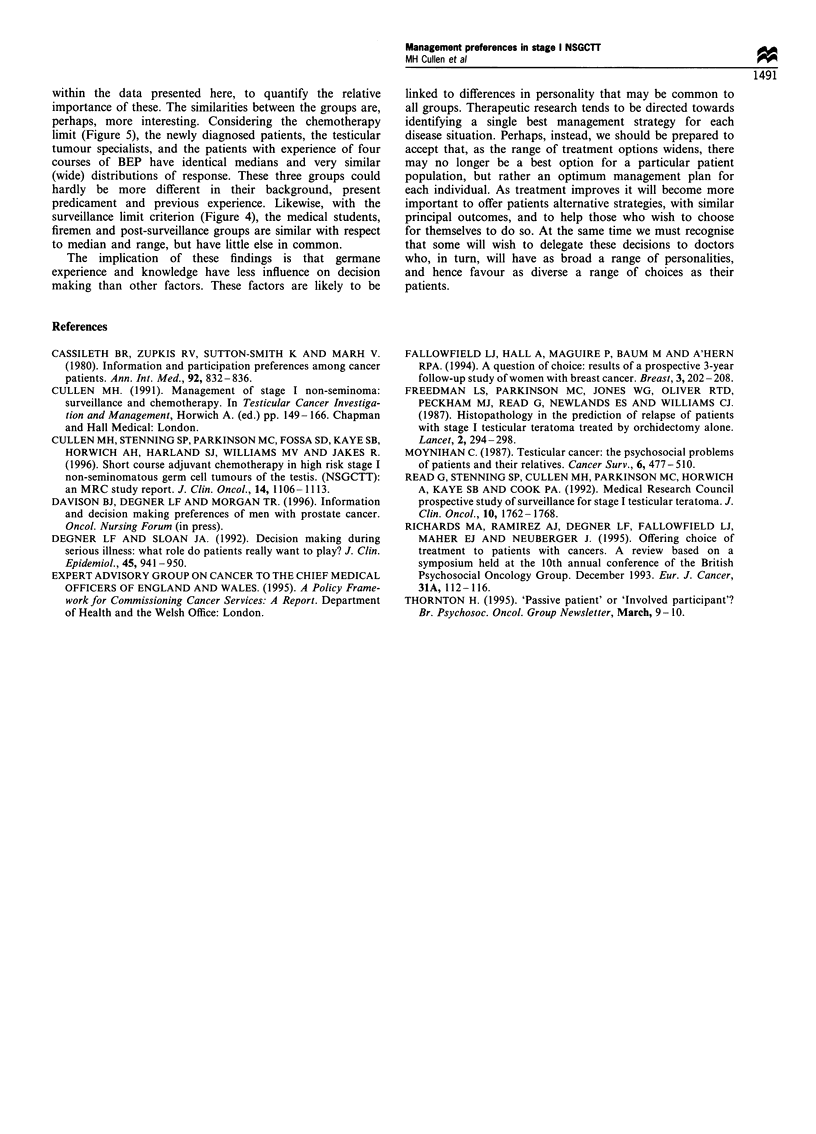# Management preferences in stage I non-seminomatous germ cell tumours of the testis: an investigation among patients, controls and oncologists.

**DOI:** 10.1038/bjc.1996.570

**Published:** 1996-11

**Authors:** M. H. Cullen, L. J. Billingham, J. Cook, C. M. Woodroffe

**Affiliations:** Birmingham Oncology Centre, University Hospital, UK.

## Abstract

Increasingly, treatment choices leading to the same survival outcome can be offered to cancer patients (e.g. mastectomy or conservative surgery in early breast cancer). Two approaches available for post-orchidectomy, stage I patients with non-seminomatous germ cell tumours of the testis (NSGCTT), particularly those at high risk of relapse, include immediate adjuvant chemotherapy (two courses) or surveillance, with chemotherapy (typically four courses) given only on relapse. The aim of this study was to investigate which approach patients prefer. Questionnaires were given to newly diagnosed NSGCTT patients, to patients with previous experience of the two options and to non-cancer controls, including specialist testicular tumour oncologists. Participants were asked to choose between immediate chemotherapy, surveillance or for the doctor to decide, at recurrence risk levels ranging from 10% to 90%. Questionnaires were returned by 207 subjects in nine different groups. The risk thresholds at which subjects' management preference changed, within apparently homogeneous groups, varied greatly, although at least one subject in each group selected adjuvant chemotherapy at the lowest (10%) level of risk. Subjects tended to favour options of which they had previous experience. Cancer patients wanted the doctor to decide more frequently than controls. The wide variability observed makes it difficult to predict which option an individual will select. Personality factors and personal circumstances, other than specific experience and knowledge, are obviously influential. Many patients would prefer their doctor to decide, but variability among oncologists is as great as that among their patients.


British Journal of Cancer (1996) 74, 1487-1491
© 1996 Stockton Press All rights reserved 0007-0920/96 $12.00

### Management preferences in stage I non-seminomatous germ cell tumours of the testis: an investigation among patients, controls and oncologists

MH Cullen[1], LJ Billingham[2], J Cook[2] and CM Woodroffe[2]

[1]*Birmingham Oncology Centre, University Hospital, Birmingham NHS Trust, Birmingham B15 2TH, UK;* [2]*CRC Trials Unit, Institute for Cancer Studies, University of Birmingham, UK.*

**Summary** Increasingly, treatment choices leading to the same survival outcome can be offered to cancer patients (e.g. mastectomy or conservative surgery in early breast cancer). Two approaches available for post-orchidectomy, stage I patients with non-seminomatous germ cell tumours of the testis (NSGCTT), particularly those at high risk of relapse, include immediate adjuvant chemotherapy (two courses) or surveillance, with chemotherapy (typically four courses) given only on relapse. The aim of this study was to investigate which approach patients prefer. Questionnaires were given to newly diagnosed NSGCTT patients, to patients with previous experience of the two options and to non-cancer controls, including specialist testicular tumour oncologists. Participants were asked to choose between immediate chemotherapy, surveillance or for the doctor to decide, at recurrence risk levels ranging from 10% to 90%. Questionnaires were returned by 207 subjects in nine different groups. The risk thresholds at which subjects' management preference changed, within apparently homogeneous groups, varied greatly, although at least one subject in each group selected adjuvant chemotherapy at the lowest (10%) level of risk. Subjects tended to favour options of which they had previous experience. Cancer patients wanted the doctor to decide more frequently than controls. The wide variability observed makes it difficult to predict which option an individual will select. Personality factors and personal circumstances, other than specific experience and knowledge, are obviously influential. Many patients would prefer their doctor to decide, but variability among oncologists is as great as that among their patients.

**Keywords:** stage I non-seminomatous germ cell tumour of the testis; adjuvant chemotherapy; surveillance; management preference

Following surgical removal of the primary tumour, patients with stage I non-seminomatous germ cell tumours of the testis (NSGCTT-'testicular teratoma') have an overall risk of developing metastatic disease of 30% (Cullen, 1991). However, a subgroup of these patients, which can be identified histologically, has an increased risk of distant recurrence of 50% (Freedman *et al.*, 1987). A recent MRC study has demonstrated that two courses of chemotherapy, administered not more than 6 weeks after surgery, will increase relapse-free and long-term survival rates to more than 98% in this 'high-risk' subgroup (Cullen *et al.*, 1996). Stage I cases who are managed in a surveillance programme, with chemotherapy (typically four courses) reserved for those patients who relapse, enjoy an identical long-term survival rate (Read *et al.*, 1992). Thus, there is a choice of two different management approaches for high-risk stage I teratoma patients; either immediate adjuvant chemotherapy, or the surveillance programme with chemotherapy only given on relapse. Both approaches lead to the same, excellent, survival prospects for this group of patients, but their attendant shortcomings are different.

It is clear that some patients experience anxiety as a result of the uncertainty of surveillance (Moynihan, 1987). Prompted by this, we sought to investigate which management approach patients prefer. Newly diagnosed teratoma patients were asked to imagine risks of recurrence ranging from 10% to 90% and then to choose between: immediate adjuvant chemotherapy (AC); surveillance (S); or for the doctor to make the decision (DD), at each risk level.

We also presented the same hypothetical scenario to different groups of controls, namely: subjects with the 'ultimate' experience, i.e. patients with testicular teratoma who have been through a surveillance programme, and others who have been treated with chemotherapy; those who might be considered to have the 'ultimate' knowledge, i.e. specialist testicular tumour oncologists; and two other groups of non-cancer controls.

### Subjects and methods

Nine separate groups, which might reasonably be expected to respond differently owing to varying experience or knowledge, were studied:

1. Newly diagnosed patients with stage I NSGCTT (New; $n=18$). For this group it was a 'real-life' situation. Patients completed the questionnaire before their individual recurrence risk had been established.
2. Patients with stage I NSGCTT who had completed an 18 month surveillance programme without recurrence (Post-surv; $n=11$).
3. Patients with stage I NSGCTT who were under surveillance (for a median of 7 months) within 18 months of diagnosis (Surv; $n=16$).
4. Patients with stage I NSGCTT who had completed two courses of adjuvant BEP chemotherapy, having been diagnosed as high-risk stage I NSGCTT (AC; $n=6$).
5. Patients with metastatic NSGCTT who had completed four courses of BEP chemotherapy with no evidence of recurrence (BEP4; $n=31$).
6. Patients with stage I NSGCTT who had experienced surveillance, but then relapsed and undergone BEP chemotherapy and were in remission, presumed cured (Relsurv; $n=9$).
7. Non-cancer controls-1; medical students having just had a lecture on NSGCTT (Students; $n=56$).
8. Non-cancer controls-2; firemen (Firemen; $n=37$). All the available officers at one fire station were selected.
9. Non-cancer controls-3; Specialist testicular tumour oncologists (Oncol; $n=18$). These were all the oncologist members of the MRC testicular tumour working party responsible for managing the vast majority of cases in the UK.

A specifically trained oncology nurse was responsible for administering a detailed information sheet and questionnaire to

Correspondence: MH Cullen
Received 8 March 1996; revised 20 May 1996; accepted 21 May 1996

all those testicular teratoma patients attending the outpatient clinic of one consultant (MC), over a period of a year. The information sheet describes the background and management options clearly, including likely short- and long-term side-effects. The oncologists were sent the same information sheet and questionnaire through the post. The two other control groups, medical students and firemen, were approached via a group lecture on the disease, followed by administration of the information sheet and questionnaire. The first part of the questionnaire recorded demographic information such as age, marital status, number of children, plans to have children and experience of serious illness. The second part of the questionnaire then asked which management approach the individual would choose given the hypothetical risks of secondaries of 10%, 20%, 30%, 40%, 50%, 70% and 90%. In the current state of knowledge it is not possible to distinguish groups with a recurrence risk of 70% or 90%, but these were included as an internal control to help assess how well the participants had understood to exercise.

Intuitively, surveillance is likely to be more popular at lower levels of risk, and chemotherapy at the upper levels. Making a firm selection for oneself is likely to be easiest at the extremes of risk with the third option (i.e. for the doctor to decide) being selected in the middle ranges.

The date were investigated in two ways. Firstly, for each group, the number of participants selecting the three options was plotted against each recurrence risk as line graphs. These are referred to as 'group trends'. Secondly, to observe trends within subjects and also to facilitate comparisons between groups, three response variables of interest were derived from the questionnaire data. These are called 'subject trends':

(1) Surveillance limit (see example below). This is the upper limit of range of risk for which the subject chooses the surveillance option. A subject is assumed to want surveillance right up to the point at which he chooses the chemotherapy or doctor decide option.
(2) Chemotherapy limit. This is the lower limit of range of risk for which the subject chooses the chemotherapy option.
(3) Uncertainty range. This is the range of risk between which the subject prefers the doctor to decide. For those subjects who never select this option (1) and (2) will be the same.

Example

| 10 | 20 | 30 | 40 | 50 | 70 | 90 |
|---|---|---|---|---|---|---|
| S | S | DD | DD | AC | AC | AC |

Surveillance limit (at 30) — Chemotherapy limit (at 50)

Uncertainty range (30–50)

The data for (1) and (2) are illustrated using box and whisker plots. These give the whole range of limits selected by that group (the whiskers), the middle 50% of values (in the box) and the median. The data for (3) are illustrated using a plot which shows, for each group, the median lower and upper risk levels of the uncertainty range. The effect of age on response in the whole group of participants was investigated using Spearman's correlation coefficient, while the effect of marital status and having children was investigated using Wilcoxon tests. The difference in response between male and female medical students was investigated using a Wilcoxon test. A 5% level of statistical significance was used.

### Results

Questionnaires were completed by 207 subjects from nine different populations. The presence of the research nurse was felt to have contributed to the high compliance rate of 98%. The responses given by five individuals (from a variety of groups) clearly suggested that they had misunderstood the questionnaire and so they were eliminated from the analysis. Table I shows the background information for the 202 individuals.

### *Group trends*

The group trends are shown for three selected groups of subjects – newly diagnosed cases (Figure 1), patients who had personal experience of four courses of chemotherapy (Figure 2) and patients who have previous experience of surveillance (Figure 3). In all groups, as the level of risk increased, the number opting for adjuvant chemotherapy increased and the number choosing surveillance decreased.

The new patients and post-chemotherapy patients responded to the hypothetical scenario in a very similar fashion, except that the level of risk above which the preference for chemotherapy became clear was higher in the new patients compared with the post-chemotherapy patients (30% and 20% respectively). In both these groups the number choosing the doctor decide option was greatest at the lower levels of risk, peaking at the 20% risk.

The picture for patients who have previous experience of surveillance is rather different. In this group, the level of risk above which the preference for chemotherapy was clear was higher, at 50%, and the number choosing the doctor decide option was greatest at the 40% and 50% levels of risk.

In all three groups of patients, approximately 25% selected adjuvant chemotherapy even at the lowest (10%) level of risk.

### *Subject trends*

*Surveillance limit* The surveillance limit is defined as the upper limit of range of risk for which the subject chooses the surveillance option (Figure 4).

The range of choice within each group is wide. At least one individual in each group preferred to avoid surveillance even at the lowest 10% risk of recurrence. On the other hand, two oncologists and one BEP4 patient preferred surveillance throughout the range, including the highest risk of 90%. The upper surveillance limit for all other groups was 70%, except those cases with high-risk stage I NSGCTT, who had been managed with adjuvant chemotherapy (AC), in whom it was 50%.

The median surveillance limit was lowest (10%) for those patients who had previously relapsed on surveillance and the adjuvant chemotherapy group. The median limit was 20% for the new stage I patients and those previously treated with four courses. The highest median surveillance limit (40%), i.e. those apparently most in favour of this policy, was observed

**Table I** Background information

| | n | *Number of women* | *Median age (range)* | *Number (%) married/ cohabiting* | *Number (%) with no children* |
|---|---|---|---|---|---|
| New | 18 | – | 35 (24–44) (2 unknown) | 10 (56%) | 8 (44%) |
| Postsurv | 11 | – | 41 (21–57) | 5 (46%) | 3 (27%) |
| Surv | 16 | – | 31 (23–60) [1 unknown] | 8 (50%) | 11 (69%) |
| AC | 6 | – | 30 (24–53) [1 unknown) | 3 (50%) | 1 (17%) |
| BEP4 | 31 | – | 31 (21–49) (3 unknown) | 19 (61%) | 15 (48%) |
| Relsurv | 9 | – | 31 (23–37) [2 unknown) | 4 (67%) (3 unknown) | 2 (33%) (3 unknown) |
| Students | 56 | 32 (58%) (1 unknown) | 22 (21–29) (1 unknown) | 3 (5%) | 56 (100%) |
| Firemen | 37 | – | 42 (30–52) | 34 (94%) (1 unknown) | 4 (11%) (1 unknown) |
| Oncol | 18 | – | – | – | – |

**Figure 1** Number of patients selecting each option at specified risk levels ($n = 18$).

**Figure 2** Number of post-chemotherapy patients (BEP4) selecting each option at specified risk levels ($n = 31$).

**Figure 3** Number of post-surveillance patients selecting each option at specified risk levels ($n = 11$).

in the patients on surveillance and the oncologists. In the remaining three groups (including the students and firemen) the median limit for choosing surveillance was 30%.

*Chemotherapy limit* The chemotherapy limit is the lower limit of range of risk for which the subject chooses the chemotherapy option (Figure 5).

Again the range is very wide among all groups extending from 10% to >90% in all but three. Each group has at least one subject choosing chemotherapy at the lowest (10%) recurrence risk, and, with the exception of the students, the adjuvant chemotherapy group and the new patients, at least

**Figure 4** Surveillance limit for each group – box and whisker plot showing median, interquartile and full ranges. Key to groups in text. Median shown by heavy line, interquartile range by box and full range by whiskers.

**Figure 5** Chemotherapy limit for each group – box and whisker plot showing median, interquartile and full ranges. Key to groups in text. Median shown by heavy line, interquartile range by box and full range by whiskers.

one subject did not select adjuvant chemotherapy even when the risk of secondaries was as high as 90%. The variability is such that even the middle 50% of limits chosen (i.e. the box part of Figure 5) lie in ranges of 30% or more.

The *median* recurrence risk at which subjects choose adjuvant chemotherapy is lowest (25%) for those who have had that management policy (AC), is 30% for the medical students and firemen, 40% for the oncologists, newly diagnosed stage I and BEP4 patients and as high as 70% for patients on surveillance or who have completed surveillance.

*Uncertainty range* The uncertainty range is defined as the range of risk within which the subject prefers his doctor to decide which treatment schedule to follow.

The proportion of participants wanting the doctor to decide on management for at least one of the decision points is shown in Table II. Not surprisingly, the control groups have the lowest proportions of respondents making this choice, particularly the oncologists and medical students with fewer than 10%. In the other groups, all of whom have or have had testicular cancer, between 33% and 64% opted for the decision to be made by the doctor at one or more risk levels.

The range of uncertainty was narrow and identical (30–40%) for the oncologists and medical students (Figure 6). The other groups were uncertain over a broader range,

**Table II** Proportion of each group who wanted the doctor to decide at some level

| | *Number/ total* | *Percentage* |
|---|---|---|
| New | 8/18 | 44 |
| Post-surveillance | 7/11 | 64 |
| Surveillance | 7/16 | 44 |
| Adjuvant chemotherapy | 2/6 | 33 |
| BEP4 | 12/31 | 39 |
| Relapse on surveillance | 5/9 | 56 |
| Students | 5/56 | 9 |
| Firemen | 6/37 | 16 |
| Oncol | 1/18 | 6 |

**Figure 6** Median start and end points of the uncertainty range for each group.

extending between 10% and 90%, but with notable differences in pattern between groups. The uncertainty range for new patients and those who had four courses of BEP was at a lower level than the other patient groups. The range of uncertainty was widest in the patients who relapsed on surveillance (30–90%).

### *Association of response with background information*

No significant differences were found between male and female medical students in terms of the surveillance limit ($P=0.83$) and the chemotherapy limit ($P=0.93$). For all participants, age and marital status did not appear to be associated with the surveillance limit ($P=0.83$ and $P=0.38$ respectively) or the chemotherapy limit ($P=0.29$ and 0.86 respectively). Although the result did not reach statistical significance ($P=0.06$), there was some evidence to suggest that having no children affected the surveillance limit, with those having no children choosing surveillance up to higher levels of risk compared with those who already had a family. The existence of children was not found to have an effect on the chemotherapy limit ($P=0.73$).

### Discussion

The recently published Expert Advisory Group on Cancer Report (1995) stresses the importance of giving patients clear information about treatment options. However, we know very little about how patients use such information in decision making (Richards *et al.*, 1995) and most of the available data relate to early breast cancer (Fallowfield *et al.*, 1994). Increasingly, there are therapeutic alternatives for cancer patients and choices to be made. The present study was originally conceived as an attempt to determine which, of two treatment options with the same outcome, would be preferred by the majority of patients with testicular teratoma. Control groups were included to test the validity of the questionnaire and information sheet. The responses resulting from the questionnaire have proved to be more interesting and thought provoking. They provide an insight into the degree to which patients are able to comprehend, and use, statistical information; how far they wish to be involved in the decision making process and to what extent related experience and knowledge may influence choice.

It was felt that the inappropriate responses of 5 of the 207 subjects (3%; four patients, one control) were an indication that they had misunderstood the exercise. (These subjects had opted for chemotherapy at the lower risk levels and had also chosen surveillance at the upper levels.) This surprisingly low failure rate was probably due to the presence of a trained nurse specialist to administer and explain the questionnaire to all but the oncologist group, and to the use of carefully worded information sheet which had been piloted before the main study.

A number of other studies of patient participation in decision making clearly show that many patients prefer key decisions to be made by the doctor (Cassileth *et al.*, 1980; Davison *et al.*, 1996). In the present study very few participants in the non-cancer control groups opted for the doctor to decide, and, in particular, the medical students and oncologists had very narrow limits of uncertainty. The other non-cancer control group, the firemen, have a slightly higher tendency to delegate the decision to the doctor than the medical students, but much less than the only other group with no experience of testicular cancer and its treatment (the newly diagnosed patients). The patients, in contrast, quite frequently opted for the doctor to decide – even those patients who had first-hand experience of at least one of the management options. This accords well with the findings of others that individuals seem to have more difficulty making decisions concerning cancer treatment when it applies to them, than when it is purely hypothetical (Degner and Sloan, 1992). However, in the present study it was essentially a hypothetical scenario for the patient groups who had completed treatment. These, too, frequently selected the doctor decide option. To what extent this reflects choice which is conditioned by the submissive patient role (Thornton, 1995), or to a more valid relinquishing of autonomy based perhaps on trust in the physician, cannot be assessed with the data discussed here.

The most striking outcome of this study is the wide variability of response within groups, with a greater consensus with regard to surveillance than there is for selecting chemotherapy. Interestingly, taking both criteria together, the group with the widest range of response is the specialist oncologists. Hence, 'ultimate knowledge' does not focus choice within narrow ranges; if anything, it has the opposite effect.

To examine the roots of this variability, it would be necessary to evaluate and compare personality traits, particularly those involved in influencing risk assessment, *across* the groups, in an attempt to detect parallels. The results described here were unexpected and hence this exercise was not undertaken. Studies of patient choice in the future should include this component.

As well as parallels, there are obvious differences between the groups. The small group of cases who had been managed with adjuvant chemotherapy is the group with the lowest median minimum risk at which that option is selected and are, therefore, apparently most in favour of this policy. Conversely, the group with previous experience of surveillance is the one requiring the highest recurrence risk before choosing chemotherapy. There are two possible explanations, which are not mutually exclusive: firstly, the patients may have been content with the option they have experienced and so would choose it again; secondly, the patients may select that option because it was how they were managed and they are reluctant to contemplate missing an alternative that was not offered, but might have been preferable. It is impossible, within the data presented here, to quantify the relative importance of these. The similarities between the groups are, perhaps, more interesting. Considering the chemotherapy limit (Figure 5), the newly diagnosed patients, the testicular tumour specialists, and the patients with experience of four courses of BEP have identical medians and very similar (wide) distributions of response. These three groups could hardly be more different in their background, present predicament and previous experience. Likewise, with the surveillance limit criterion (Figure 4), the medical students, firemen and post-surveillance groups are similar with respect to median and range, but have little else in common.

The implication of these findings is that germane experience and knowledge have less influence on decision making than other factors. These factors are likely to be linked to differences in personality that may be common to all groups. Therapeutic research tends to be directed towards identifying a single best management strategy for each disease situation. Perhaps, instead, we should be prepared to accept that, as the range of treatment options widens, there may no longer be a best option for a particular patient population, but rather an optimum management plan for each individual. As treatment improves it will become more important to offer patients alternative strategies, with similar principal outcomes, and to help those who wish to choose for themselves to do so. At the same time we must recognise that some will wish to delegate these decisions to doctors who, in turn, will have as broad a range of personalities, and hence favour as diverse a range of choices as their patients.

### References

CASSILETH BR, ZUPKIS RV, SUTTON-SMITH K AND MARH V. (1980). Information and participation preferences among cancer patients. *Ann. Int. Med.*, **92,** 832–836.

CULLEN MH. (1991). Management of stage I non-seminoma: surveillance and chemotherapy. In *Testicular Cancer Investigation and Management*, Horwich A. (ed.) pp. 149–166. Chapman and Hall Medical: London.

CULLEN MH, STENNING SP, PARKINSON MC, FOSSA SD, KAYE SB, HORWICH AH, HARLAND SJ, WILLIAMS MV AND JAKES R. (1996). Short course adjuvant chemotherapy in high risk stage I non-seminomatous germ cell tumours of the testis. (NSGCTT): an MRC study report. *J. Clin. Oncol.*, **14,** 1106–1113.

DAVISON BJ, DEGNER LF AND MORGAN TR. (1996). Information and decision making preferences of men with prostate cancer. *Oncol. Nursing Forum* (in press).

DEGNER LF AND SLOAN JA. (1992). Decision making during serious illness: what role do patients really want to play? *J. Clin. Epidemiol.*, **45,** 941–950.

EXPERT ADVISORY GROUP ON CANCER TO THE CHIEF MEDICAL OFFICERS OF ENGLAND AND WALES. (1995). *A Policy Framework for Commissioning Cancer Services: A Report*. Department of Health and the Welsh Office: London.

FALLOWFIELD LJ, HALL A, MAGUIRE P, BAUM M AND A'HERN RPA. (1994). A question of choice: results of a prospective 3-year follow-up study of women with breast cancer. *Breast*, **3,** 202–208.

FREEDMAN LS, PARKINSON MC, JONES WG, OLIVER RTD, PECKHAM MJ, READ G, NEWLANDS ES AND WILLIAMS CJ. (1987). Histopathology in the prediction of relapse of patients with stage I testicular teratoma treated by orchidectomy alone. *Lancet*, **2,** 294–298.

MOYNIHAN C. (1987). Testicular cancer: the psychosocial problems of patients and their relatives. *Cancer Surv.*, **6,** 477–510.

READ G, STENNING SP, CULLEN MH, PARKINSON MC, HORWICH A, KAYE SB AND COOK PA. (1992). Medical Research Council prospective study of surveillance for stage I testicular teratoma. *J. Clin. Oncol.*, **10,** 1762–1768.

RICHARDS MA, RAMIREZ AJ, DEGNER LF, FALLOWFIELD LJ, MAHER EJ AND NEUBERGER J. (1995). Offering choice of treatment to patients with cancers. A review based on a symposium held at the 10th annual conference of the British Psychosocial Oncology Group. December 1993. *Eur. J. Cancer*, **31A,** 112–116.

THORNTON H. (1995). 'Passive patient' or 'Involved participant'? *Br. Psychosoc. Oncol. Group Newsletter*, **March,** 9–10.